# Performance of LDBio *Aspergillus* WB and ICT Antibody Detection in Chronic Pulmonary Aspergillosis

**DOI:** 10.3390/jof7040311

**Published:** 2021-04-18

**Authors:** Anna Rozaliyani, Findra Setianingrum, Sresta Azahra, Asriyani Abdullah, Ayu Eka Fatril, Harmi Rosianawati, Erlina Burhan, Diah Handayani, Arief Riadi Arifin, Jamal Zaini, Mulyati Tugiran, Robiatul Adawiyah, Ridhawati Syam, Heri Wibowo, Retno Wahyuningsih, Chris Kosmidis, David W Denning

**Affiliations:** 1 Department of Parasitology, Faculty of Medicine, Universitas Indonesia, Jakarta 10430, Indonesia; findra.s88@gmail.com (F.S.); asriyaniabdullah1464@gmail.com (A.A.); ayufatril34@gmail.com (A.E.F.); dramulyati@yahoo.co.id (M.T.); bundaadah@gmail.com (R.A.); ridhawatia@yahoo.com (R.S.); bowoheri04@gmail.com (H.W.); retnet2002@gmail.com (R.W.); 2 Pulmonary Mycosis Centre, Jakarta 10430, Indonesia; erlina_burhan@yahoo.com (E.B.); diahzulfitri@yahoo.com (D.H.); riadiarifin@yahoo.com (A.R.A.); jamalzaini@gmail.com (J.Z.); 3 Magister Program of Biomedical Sciences, Faculty of Medicine, Universitas Indonesia, Jakarta 10430, Indonesia; sresta.azahra@gmail.com; 4 Department of Pulmonology and Respiratory Medicine, Faculty of Medicine, Universitas Indonesia, Persahabatan National Respiratory Referral Hospital, Jakarta 13230, Indonesia; harmirosi520@gmail.com; 5 MH Thamrin Hospital, Jakarta 10440, Indonesia; 6 Department of Parasitology, Faculty of Medicine, Universitas Kristen, Jakarta 13530, Indonesia; 7 Manchester Academic Health Science Centre, Division of Infection, Immunity and Respiratory Medicine, Faculty of Biology, School of Biological Sciences, Medicine and Health, University of Manchester, Manchester M23 9LT, UK; chris.kosmidis@manchester.ac.uk (C.K.); ddenning@manchester.ac.uk (D.W.D.)

**Keywords:** chronic pulmonary aspergillosis, Western blot, immunochromatography

## Abstract

The detection of *Aspergillus* antibody has a key role in the diagnosis of chronic pulmonary aspergillosis. Western blot (WB) and immunochromatography (ICT) lateral flow detection of *Aspergillus* antibody can be used as confirmatory and screening assays but their comparative performance in TB patients is not known. This study investigated the performance of these assays among 88 post-tuberculosis patients with suspected CPA. Sensitivity, specificity, receiver operating curve (ROC), area under-curve (AUC) and the agreement between two assays were evaluated. Both WB and ICT showed good sensitivity (80% and 85%, respectively) for detection of *Aspergillus* antibodies. Substantial agreement (0.716) between these assays was also obtained. The highest AUC result (0.804) was achieved with the combination of WB and ICT. The global intensity of WB correlated with the severity of symptoms in CPA group (*p* = 0.001). The combination of WB and ICT may increase specificity in CPA diagnosis.

## 1. Introduction

Chronic pulmonary aspergillosis is the most common manifestation of aspergillosis in patients with pre-existing lung conditions such as tuberculosis [1]. Persistent and progressive pulmonary symptoms and lung tissue destruction occur in CPA with a high 5-year mortality rate, ranging from 50% to 85% [2,3]. A recent study from Indonesia reported that 13% of patients developed CPA at the end of TB therapy [4].

Accurate CPA diagnosis is still considered a challenging issue for clinicians [5]. *Aspergillus* antibody measurement is the key diagnostic of CPA since symptoms and radiological appearances of CPA overlap with TB and other fungal infections [6]. Commercially available *Aspergillus* antibody serological assays include immunoprecipitation detection (IPD) (also in house assays used), enzyme-linked immunosorbent assay (ELISA), complement fixation, immunoblot, hemagglutination (which has poor performance), Western blot and immunochromatography (ICT) lateral flow assay [7].

The *Aspergillus* Western blot (WB) IgG kit uses immunoblotting to detect at least two of four key antibodies for CPA diagnosis. The number and intensity of bands varies in different patients. Previous studies reported good performance of this assay among various clinical presentations of aspergillosis [8,9]. However, there is no study of WB in a population with high risk of CPA such as post-tuberculosis patients. We recently reported the use of ICT in post-tuberculosis patients with 80% sensitivity and 70% specificity [10]. Unlike ICT, which is a qualitative method, the WB is a semi-quantitative antibody detection assay which might help in quantifying *Aspergillus* IgG where automated ELISA is not available. Very high baseline *Aspergillus* IgG titers reflect a better clinical response than lower levels [11,12]. A fall in antibody concentration reflects response to antifungal therapy and rising levels were observed after discontinuing therapy [13]. Therefore, this study aimed to compare the performances of the WB and ICT for CPA diagnosis in Indonesia. The findings are applicable to other limited resource countries.

## 2. Materials and Methods

Sera from 70 post-TB patients collected from April 2019 to February 2020 were tested at the Mycology Laboratory, Faculty of Medicine, Universitas Indonesia in Jakarta by ICT as previously described [10] followed by WB. The patients were adults (>18 years) who presented to a respiratory clinic (Persahabatan National Respiratory Referral Hospital, Jakarta, Indonesia) with symptoms following TB therapy. An additional 18 sera of post-TB patients were tested by ICT and WB from the sera of adults (>18 years) who presented to a respiratory clinic (Persahabatan National Respiratory Referral Hospital, Jakarta, Indonesia and MH Thamrin Hospital, Jakarta, Indonesia) following TB therapy. The Ethics Committee of the Faculty of Medicine, Universitas Indonesia approved the study (ND 071/UN2.F1/ETIK/PPM.00.02/2021). All patients provided consent.

Patients were divided into CPA and non-CPA groups. Those in the CPA group fulfilled all these criteria: (1) presence of at least one of the following pulmonary symptoms for ≥3 months: cough, hemoptysis, dyspnea, fatigue and/or chest pain; (2) evidence of *Aspergillus* spp. from sputum culture, (3) cavitation and/or fungal ball from radiological findings suggestive of CPA and (4) negative GenXpert TB and/or negative acid-fast bacilli (AFB) smear. Chest radiology interpretations were assessed by a senior radiologist from the local hospital. The definition of CPA was based on Denning et al. with some modifications [5]. We did not include detection of *Aspergillus* antibody test in our diagnosis criteria because we wanted to compare the diagnostic performance of the serology tests (WB and ICT) and the quantitative *Aspergillus* IgG test is not available in Indonesia. A single induced sputum from every patient was cultured on Sabouraud dextrose agar with high volume cultures [14]. Sputum inductions were performed with 3% NaCl solution according to the hospital protocol.

Detection of *Aspergillus* IgG was performed using Western blot (LDBio Diagnostics, Lyon, France). Meanwhile, the *Aspergillus* IgG and IgM presence was tested using ICT (LDBio Diagnostics, Lyon, France). All tests were run according to manufacturer’s guidance. ICT results were reported as a qualitative result (positive or negative). Meanwhile, WB results were reported as weak positive, strong positive, or negative, number of bands and global intensity, as described previously [8].

Statistical analysis was performed using SPSS version 25 (SPSS Inc., Chicago, IL, USA). Fisher exact test, chi-square and student *t*-test were use for the descriptive statistics. We utilised pre-existing data from our previous study [10] to perform statistical analyses of clinical, radiological and laboratory variables. The agreement between ICT and WB was evaluated using Cohen’s Kappa coefficient. McNemar’s test was used to compare the sensitivity and specificity between these two assays [15]. Receiver operating curve (ROC) analyses were assessed and area under-curve (AUC) values with 95% confidence intervals were reported for WB, ICT and combinations of these two platforms.

## 3. Results

### 3.1. Patients and Clinical Characteristics

Eighty-eight patients with suspected recurrent pulmonary TB were enrolled in the study. Eighteen (20%) patients met criteria for CPA (Table 1). The most frequent symptom was fatigue (41%, *n* = 36), followed by dyspnea (38%, *n* = 33), cough (33%, *n* = 29), hemoptysis (28%, *n* = 25) and chest pain (21%, *n* = 18). Infiltrates (66%, *n* = 58), cavitation (57%, *n* = 50), bronchiectasis (36%, *n* = 32) and pleural thickening (22%, *n* = 19) were commonly observed in chest radiology. CT scan were available in 53% (*n* = 47) patients, while the rest of the patients had chest x-ray. Diabetes mellitus appeared to be the most prevalent chronic disease in our study. The median time from history of TB therapy before recruitment was 8.5 (range: 1–244) months.

### 3.2. Culture Results

The most common species isolated was *Aspergillus fumigatus* (*n* = 37, 42%), followed by *A. niger* (*n* = 22, 25%) and *A. flavus* (*n* = 2, 2%). Among 20 patients in the CPA group, 15 patients had positive *A. fumigatus* cultures, six of which were mixed with *A. niger*. The remaining five CPA patients only grew *A. niger* in their culture.

Twenty-three patients (68%) had positive *Aspergillus* culture from 34 patients with positive WB tests. The same number of patients (*n* = 23, 64%) had positive *Aspergillus* culture from 36 patients with positive ICT tests. However, these 23 patients were not exactly the same patients from both groups. Twenty patients grew *Aspergillus* and had positive tests for both WB and ICT tests.

### 3.3. Western Blot and ICT Results

The strong positive and weak positive rate of WB among 88 patients were 23.1% (*n* = 23) and 12.5% (*n* = 11), which together result in 39% (*n* = 34) positives. Of the 20 sera tested in the CPA group, 16 showed positive results by WB with 80% sensitivity (Table 2). In the non-CPA group, 50 of the 68 sera showed negative results by WB with 73.5% specificity. There was significant difference of WB positive results in CPA (*n* = 16) and non-CPA (*n* = 18) group (80% vs. 26%, *p* < 0.001).

**Figure 1:**
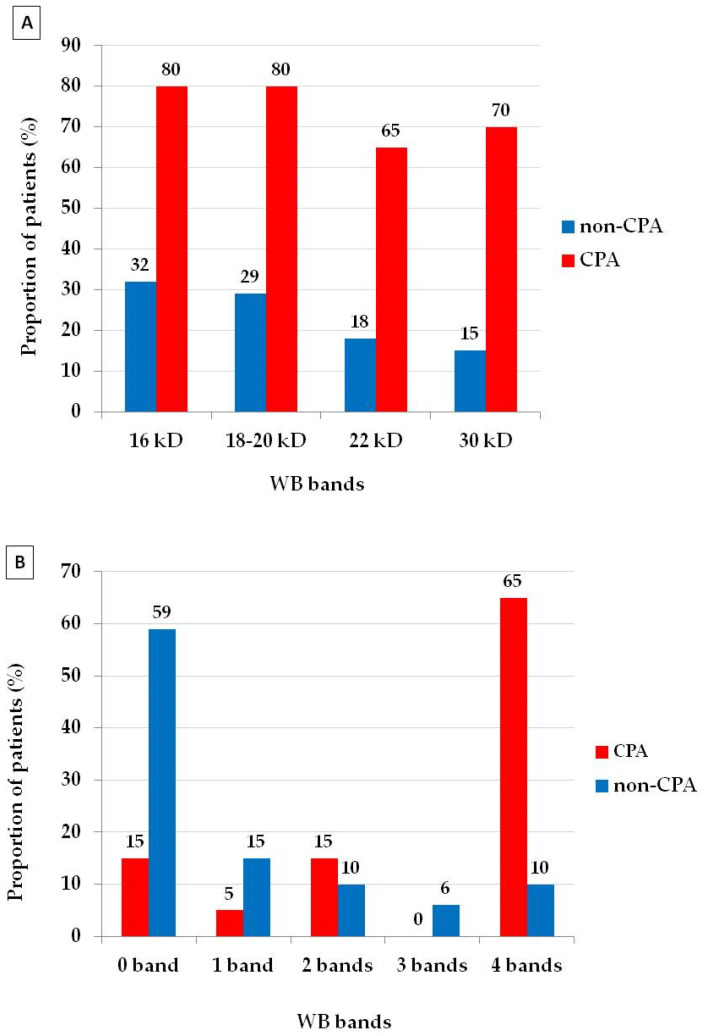
The distribution of *Aspergillus* WB banding profile in chronic pulmonary aspergillosis (CPA) and non-CPA groups. (**A**) The CPA group had a higher proportion of all four bands (16, 18–20, 22 and 30 kDa) compared to the non-CPA group (*p* < 0.001 in all comparisons). (**B**) The number of patients with 4 bands was significantly higher (*p* < 0.001) in the CPA group (65%) compare to the non-CPA group (10%).

The 16 and 18–20 kDa WB bands were the most prevalent (80%) bands appearing in the CPA group, while the 16 kDa WB band was the most common (32%) band in the non-CPA group (Figure 1). The proportion of all four WB bands (80% vs. 32% for 16 kDa, 80% vs. 29% for 18–20 kDa, 65% vs. 18% for 22 kDa and 70% vs. 15% for 30 kDa) was significantly higher in the CPA group than in the non-CPA group (*p* < 0.001 in all of the bands). Patients in the non-CPA group had lower (*p* < 0.001) median global intensity (median = 0, IQR (0–3.75)) than those in the CPA group (median 10.5, IQR (3.25–14)) (Table 3). Number of symptoms in the CPA group (median = 2, IQR (2–3)) was higher (*p* = 0.005) than in the non-CPA group (median = 1, IQR (0–2)). The WB global intensity showed correlation with the number of symptoms in the CPA group (*p* = 0.001), but not in the non-CPA group (*p* = 0.752).

**Figure 2:**
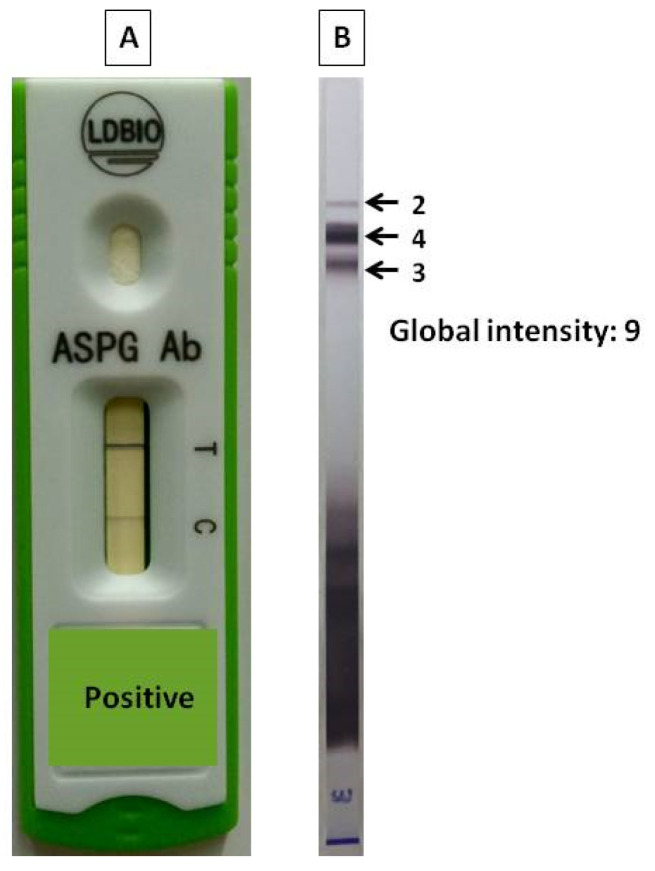
Examples of ICT and WB results from a CPA patient. (**A**) ICT positive result. (**B**) WB positive result with global intensity: 9.

ICT results were classified as positive and negative results. Thirty-six patients (41%) had positive results and 52 (59%) patients had negative results. Of the 20 sera tested in CPA group, 17 showed positive results by ICT with 85% sensitivity. In the non-CPA group, 49 of the 68 sera showed negative results by ICT with 72.1% specificity. There was significant difference of ICT positive results in CPA and non-CPA group (85% vs. 28%, *p* < 0.001). The best AUC was achieved with a combination of ICT and WB (0.804). The examples of ICT and WB results are shown in Figure 2.

**Figure 3:**
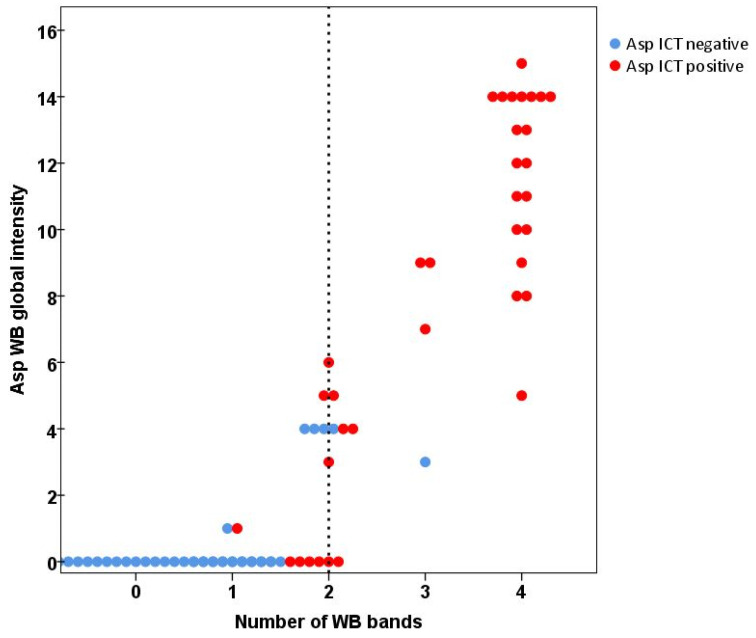
The scatterplot of *Aspergillus* IgG banding patterns in correlation with WB global intensity and Asp ICT results with minimum number of WB bands (2) indicated positive results showed as a dash line. There was a substantial agreement between ICT and WB (Cohen’s Kappa 0.716 (95% CI 0.567–0.864)).

**Figure 4:**
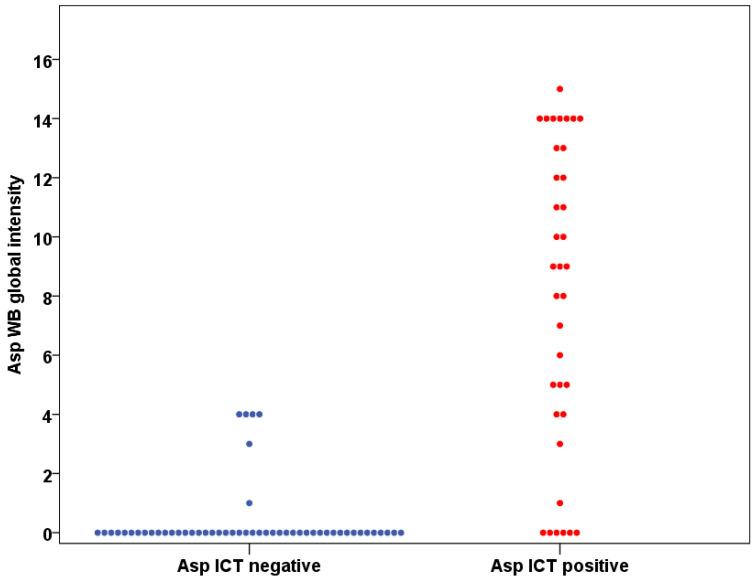
The Western blot global intensity and *Aspergillus* ICT results correlation.

Comparing WB and ICT, 76 (86%) results from the patients were in complete agreement and Cohen’s Kappa was 0.716 (95% CI 0.567–0.864), indicating substantial agreement between these two methods (Figure 3 and Figure 4). There were 12 (14%) discordant results between the WB and ICT tests. Five WB tests were positive when ICT was negative and seven WB tests were negative when ICT was positive. The sensitivity and specificity rate of both assays were not statistically different (*p* = 1 both in CPA and non-CPA patients, McNemar’s test). At least one band of *Aspergillus* IgG antibody was detected in 11 patients (12.5%) (Figure 3), as expected with a ubiquitous airborne pathogen.

## 4. Discussion

The present study has evaluated the performance of ICT and WB to diagnose CPA in post-tuberculosis patients with persistent symptoms. Both assays demonstrated good performance with 80% sensitivity for WB and 85% sensitivity for ICT. The sensitivity and specificity rate of both assays were comparable. In other studies of CPA, the sensitivity ranged from 86.7% to 93.4% for WB and from 80% to 89.8% for ICT [8,9,10]. Combination of both assays appeared to increase specificity (80.9%) and achieved the best AUC (0.804). This study is the first to report on the performance of WB in resource limited environments.

The level of agreement between these two tests in our study (Cohen’s Kappa 0.716) was higher than in the previous study (Cohen’s Kappa 0.558) [9]. The difference in Cohen’s Kappa reflected difference CPA prevalence in the population of these studies. Therefore, Cohen’s Kappa is not suitable for comparisons between different studies and population [16]. Among 12 discordant results, five patients had a weak positive WB result with negative ICT tests. All of these five patients were classified as non-CPA. Three of these five patients showed positive *Aspergillus* culture, but no radiology suggestive of CPA and/or symptoms. These might indicate *Aspergillus* colonisation in the context of bronchiectasis with WB global intensity 4 in all three patients as previous study reported sensitivity of 73.2% of WB for detection of *Aspergillus* colonisation [8]. Another two of these five patients without antibody had prominent radiology findings with multiple cavities without positive cultures or symptoms. This might represent an early phase of CPA, or more likely subtle immunodeficiency and false negative antibody tests as recently described [17]. WB might be used to detect early stages of CPA while ICT remains negative.

The difference in sensitivity between ICT and WB was caused by a discordant result in only one CPA patient. WB failed to detect the IgG in this patient with CPA symptoms (chronic cough, dyspnea and fatigue) and multiple cavities in both lungs. This patient had positive result of *A. fumigatus* culture. This may be due to the ability of ICT to detect IgM in addition to IgG, as the presence of IgM was reported in up to 50% of CPA cases [18,19,20]. Elevated IgM levels in patients after surgery for aspergilloma was detected, while the IgG existed in low level [19]. Therefore, it might be possible that in our study only IgM was detected as immunological response in CPA. It could be hypothesised that IgM was detected following a re-stimulated immune response to new and different *Aspergillus* antigens as each growth cycle of *Aspergillus* can produce various *Aspergillus* antigens [21,22]. Guo et al. reported limited diagnostic capacity of *Aspergillus* IgM for CPA diagnosis (sensitivity 58.8% and specificity 68%) [23].

This is the first study evaluating the WB banding patterns of different *Aspergillus* species since the previous study included analyses for *A. fumigatus* cases only [8]. The most common WB band in CPA with *A. fumigatus* positive culture is the 16 kD (89%, *n* = 8) and 18–20 kD (89%, *n* = 8). Meanwhile all WB bands were seen at the same rate (60%, *n* = 3) in CPA with *A. niger* positive culture. The correlation between WB global intensity and number of symptoms was revealed in our study. A recent study showed potential usefulness of the *Aspergillus* IgG titer in monitoring relapse in CPA [12]. Future study is required to explore potential use of a semi-quantitative WB global intensity for monitoring CPA patients from clinical or radiology perspectives.

There were 47 patients with both ICT and WB negative results. These 47 patients consist of 44 non-CPA patients and three CPA patients. Of these three CPA patients with negative serology, two had positive culture for *A. niger* and one had mixed-culture of *A. fumigatus* and *A. niger*. There is a possibility that *A. niger* will cause a false negative in the serology tests of ICT and WB since the antigens coated in both of the platforms were antigens from *A. fumigatus* [22]. However, we found 9 CPA patients (4 mixed with *A. fumigatus*, 5 only *A. niger*) with positive *A. niger* culture showed a positive result of ICT and WB. Future study is needed to determine the *Aspergillus* species-related antibody patterns to assist the definite diagnosis of CPA. All patients with a positive GenXpert result for *Mycobacterium tuberculosis* were classified as non-CPA in this study, but we know from our work and others that dual infection is also possible [24,25,26]. Interpretation of the radiological findings in this context is difficult and subjective.

There was no difference in the median global intensity between CPA with *A. niger* only (8, range 0–14) and CPA with *A. fumigatus* (11, range 0–14) only (*p* = 0.346). A recent study on allergic bronchopulmonary aspergillosis reported a strong correlation between IgG specific *A. fumigatus* and IgG specific *A. niger* [27]. A large seroprevalence study from Taiwan revealed high correlation (Spearman correlation coefficient: 0.942) between IgG specific *A. fumigatus* and IgG specific *A. niger* using automated ImmunoCAP systems [28]. However, there is still the probability of a different immunological response between these two species which might cause a false negative result of ICT and WB in our study. *A. niger* is an especially common cause of CPA in diabetic patients and may lead to systemic oxalosis [29,30,31]. Alternatively, the *A. niger* isolated could be an airway contaminant, obscuring infection with *A. fumigatus*, which can be difficult to grow in these patients. This study has the shortcoming of the small sample size regarding interspecies variety of IgG response between *A. fumigatus* and *A. niger*. Furthermore, our criteria for diagnosis of CPA did not include serology, only microbiology. This may have underestimated the prevalence of CPA as culture is known to be less sensitive than serology.

In conclusion, this study reports comparable performance of WB and ICT for the diagnosis of CPA in post-tuberculosis patients. The procedure of ICT is simpler and faster than WB. The WB tests require specific skills and laboratory facilities. However, WB may be used as a confirmatory test for CPA. The use of WB band profile and global intensity for early detection of CPA should be explored in a larger study.

## Figures and Tables

**Table 1:** Patient characteristics.

Variables	All (*n* = 88)	CPA (*n* = 20)	Non CPA(*n* = 68)	*p*-Value
Gender				
Male	61 (69%)	14 (70%)	47 (69%)	
Female	27 (31%)	6 (30%)	21 (31%)	0.940
Age, mean (range)	49 (18–79)	50 (28–66)	49 (18–79)	0.919
**Chronic diseases**				
Diabetes mellitus	11 (13%)	7 (39%)	4 (6%)	0.002
Hypertension	8 (9%)	1 (6%)	7 (10%)	0.676
Asthma	6 (7%)	1 (6%)	5 (7%)	1
Chronic obstructive pulmonary disease	9 (10%)	2 (11%)	7 (10%)	1
Duration of TB therapy (range), months	9 (2–26)	13 (9–17)	8 (7–9)	<0.001
TB therapy >6 months	34 (39%)	13 (65%)	21 (31%)	0.007
Time from end of TB therapy to recruitment (months), median (range)	8.5 (1–244)	5 (1–56)	9 (1–244)	0.245
Smoking history	50 (57%)	14 (70%)	36 (53%)	0.176

**Table 2:** Diagnostic performances of *Aspergillus* Western blot and immunochromatography (ICT) tests.

Test	% Sensitivity (95% CI)	% Specificity (95% CI)	AUC ROC
Western blot	80 (56.3–94.3)	73.5 (61.4–83.5)	0.768
ICT	85 (62.1–96.8)	72.1 (59.9–82.3)	0.785
Western blot + ICT	80 (56.3–94.3)	80.9 (69.5–89.4)	0.804

**Table 3:** Western blot global intensity and number of symptoms in CPA and non-CPA groups.

	All (*n* = 88)	CPA (*n* = 20)	Non-CPA (*n* = 68)	*p*-Value
WB Global intensity				
Median	0	10.5	0	<0.001
Mean (95% CI)	3.5 (2.4–4.6)	8.75 (6.1–11.4)	2 (1.1–2.8)	
Interquartile range	0–6.75	3.25–14	0–3.75	
Maximum	0	0	0	
Minimum	15	15	15	
Number of symptoms				
Median	1.5	2	1	0.005
Mean	1.6 (1.3–1.9)	2.3 (1.8–2.8)	1.4 (1.1–1.7)	
Interquartile range	0–3	2–3	0–2	
Maximum	0	0	0	
Minimum	5	5	4	
WB global intensity & number of symptoms correlation (*p*)	0.010	0.001	0.752	

## References

[1] Denning D.W., Pleuvry A., Cole D.C. (2011). Global Burden of Chronic Pulmonary Aspergillosis as a Sequel to Pulmonary Tuberculosis. Bull. World Health Organ..

[2] Kosmidis C., Denning D.W. (2015). The Clinical Spectrum of Pulmonary Aspergillosis. Thorax.

[3] Lowes D., Al-Shair K., Newton P.J., Morris J., Harris C., Rautemaa-Richardson R., Denning D.W. (2017). Predictors of Mortality in Chronic Pulmonary Aspergillosis. Eur. Respir. J..

[4] Setianingrum F., Rozaliyani A., Syam R., Adawiyah R., Tugiran M., Sari C.Y.I., Burhan E., Wahyuningsih R., Rauteema-Richradson R., Denning D.W. (2020). Evaluation and Comparison of Automated and Manual ELISA for Diagnosis of Chronic Pulmonary Aspergillosis (CPA) in Indonesia. Diagnostic Microbiol. Infect. Dis..

[5] Denning D.W., Page I., Chakaya J., Jabeen K., Jude C.M., Cornet M., Alastruey-Izquierdo A., Bongomin F., Bowyer P., Chakrabarti A. (2018). Case Definition of Chronic Pulmonary Aspergillosis in Resource-Limited Settings: Catalysing Research and Clinical Care. Emerg. Infect. Dis..

[6] Denning D.W., Cadranel J., Beigelman-Aubry C., Ader F., Chakrabarti A., Blot S., Ullmann A.J., Dimopoulos G., Lange C. (2016). Chronic Pulmonary Aspergillosis: Rationale and Clinical Guidelines for Diagnosis and Management. Eur. Respir. J..

[7] Richardson M., Page I. (2018). Role of Serological Tests in the Diagnosis of Mold Infections. Curr. Fungal Infect. Rep..

[8] Oliva A., Flori P., Hennequin C., Dubus J.C., Reynaud-Gaubert M., Charpin D., Vergnon J.M., Gay P., Colly A., Piarroux R. (2015). Evaluation of the Aspergillus Western Blot IgG Kit for Diagnosis of Chronic Aspergillosis. J. Clin. Microbiol..

[9] Stucky Hunter E., Richardson M.D., Denning D.W. (2019). Evaluation of LDBio Aspergillus ICT Lateral Flow Assay for IgG and IgM Antibody Detection in Chronic Pulmonary Aspergillosis. J. Clin. Microbiol..

[10] Rozaliyani A., Rosianawati H., Handayani D., Agustin H., Zaini J., Syam R., Adawiyah R., Tugiran M., Setianingrum F., Burhan E. (2020). Chronic Pulmonary Aspergillosis in Post Tuberculosis Patients in Indonesia and the Role of Ldbio Aspergillus Ict as Part of the Diagnosis Scheme. J. Fungi.

[11] Bongomin F., Otu A., Harris C., Foden P., Kosmidis C., Denning D.W. (2020). Risk Factors for Relapse of Chronic Pulmonary Aspergillosis after Discontinuation of Antifungal Therapy. Clin. Infect. Pract..

[12] Setianingrum F., Rautemaa-Richardson R., Shah R., Denning D.W. (2020). Clinical Outcomes of Patients with Chronic Pulmonary Aspergillosis Managed Surgically. Eur. J. Cardio Thorac. Surg..

[13] Bongomin F., Harris C., Hayes G., Kosmidis C., Denning D.W. (2018). Twelve-Month Clinical Outcomes of 206 Patients with Chronic Pulmonary Aspergillosis. PLoS ONE.

[14] Vergidis P., Moore C.B., Novak-Frazer L., Rautemaa-Richardson R., Walker A., Denning D.W., Richardson M.D. (2020). High-Volume Culture and Quantitative Real-Time PCR for the Detection of Aspergillus in Sputum. Clin. Microbiol. Infect..

[15] Kim S., Lee W. (2017). Does McNemar’s Test Compare the Sensitivities and Specificities of Two Diagnostic Tests?. Stat. Methods Med. Res..

[16] Viera A.J., Garrett J.M. (2005). Understanding Interobserver Agreement: The Kappa Statistic. Fam. Med..

[17] Hunter E.S., Wilopo B., Richardson M.D., Kosmidis C., Denning D.W. (2021). Effect of Patient Immunodeficiencies on the Diagnostic Performance of Serological Assays to Detect Aspergillus -Specific Antibodies in Chronic Pulmonary Aspergillosis. Respir. Med..

[18] Kauffman H., van der Heide S., Beaumont F., Blok H., de Vries K. (1986). Class-Specific Antibody Determintaion against Aspergillus Fumigatus by Means of the Enzyme-Linked Immunosorbent Assay. Int. Archs Allergy appl. Immun..

[19] Kostiala A.I., Stenius-aarniala B., Alanko K. (1984). Analysis of Antibodies to Aspergillus Fumigatus Antigens by Class-Specific Enzyme-Linked Immunosorbent Assay in Patients with Pulmonary Aspergillosis. Diagn. Microbiol. Infect. Dis..

[20] Weig M., Frosch M., Tintelnot K., Haas A., Groß U., Linsmeier B., Heesemann J. (2001). Use of Recombinant Mitogillin for Improved Serodiagnosis of Aspergillus Fumigatus-Associated Diseases. J. Clin. Microbiol..

[21] Bozza S., Clavaud C., Giovannini G., Beauvais A., Sarfati J., Angelo D., Perruccio K., Bonifazi P., Moretti S., Bistoni F. (2009). Immune Sensing of Aspergillus Fumigatus Proteins, Glycolipids, and Polysaccharides and the Impact on Th Immunity and Vaccination. J. Immunol..

[22] Page I.D., Richardson M., Denning D.W. (2015). Antibody Testing in Aspergillosis--Quo Vadis?. Med. Mycol..

[23] Guo Y., Bai Y., Yang C., Gu L. (2019). Evaluation of Aspergillus IgG, IgM Antibody for Diagnosing in Chronic Pulmonary Aspergillosis: A Prospective Study from a Single Center in China. Medicine.

[24] Iqbal N., Irfan M., Bin A., Zubairi S., Jabeen K., Awan S., Khan J.A. (2016). Clinical Manifestations and Outcomes of Pulmonary Aspergillosis: Experience from Pakistan. BMJ Open Respir. Res..

[25] Bekele A., Ali A., Biluts H. (2008). Surgically Treated Pulmonary Tuberculosis: Report on Cases from Tikur Anbess Hospital, Addis Ababa, Ethiopia. Ethiop. Med. J..

[26] Setianingrum F., Rozaliyani A., Adawiyah R., Syam R., Tugiran M., Sari C.Y.I., Nandipinto F., Ramnath J., Arifin A.R., Handayani D. A Prospective Longitudinal Study of Chronic Pulmonary Aspergillosis in Pulmonary Tuberculosis in Indonesia (APICAL). Thorax.

[27] Kuwabara K., Hirose M., Kato K., Yokoi T., Shiga M., Kondo R., Nakamura M., Matsunaga K. (2020). Serological Analysis of Sensitization in Allergic Bronchopulmonary Aspergillosis: A Study on Allergen Components and Interspecies Relationships. J. Asthma.

[28] Lee M., Huang H., Chen L., Yang H., Ko J., Cheng M., Chong I., Lee L., Wang J., Dimopoulos G. (2019). Seroprevalence of Aspergillus IgG and Disease Prevalence of Chronic Pulmonary Aspergillosis in a Country with Intermediate Burden of Tuberculosis: A Prospective Observational Study. Clin. Microbiol. Infect..

[29] Severo L., Londero A., Geyer G., Picon P. (1981). Oxalosis Associated with An Aspergillus Niger Fungus Ball. Report of A Case. Mycopathologia.

[30] Severo L.C., Geyer G.R., Porto N.S., Micologia S.D., Bioldgicas I.D.P., Domingos R. (1990). Pulmonary Aspergillus Intracavitary Colonization (PAIC). Mycopathologia.

[31] Severo L., Geyer G., Porto N., Wagner M., Londero A. (1997). Pulmonary Aspergillus Niger Intracavitary Colonization. Report of 23 Cases and a Review of the Literature. Rev. Iberoam. Micol..

